# Integrated Proteomics and Lipidomics Reveal That the Swarming Motility of *Paenibacillus polymyxa* Is Characterized by Phospholipid Modification, Surfactant Deployment, and Flagellar Specialization Relative to Swimming Motility

**DOI:** 10.3389/fmicb.2019.02594

**Published:** 2019-11-19

**Authors:** Suresh Poudel, Richard J. Giannone, Abigail T. Farmer, Shawn R. Campagna, Amber N. Bible, Jennifer L. Morrell-Falvey, James G. Elkins, Robert L. Hettich

**Affiliations:** ^1^Biosciences Division, Oak Ridge National Laboratory, Oak Ridge, TN, United States; ^2^Graduate School of Genome Science and Technology, The University of Tennessee, Knoxville, Knoxville, TN, United States; ^3^Chemical Sciences Division, Oak Ridge National Laboratory, Oak Ridge, TN, United States; ^4^Department of Chemistry, The University of Tennessee, Knoxville, Knoxville, TN, United States; ^5^Biological and Small Molecule Mass Spectrometry Core, The University of Tennessee, Knoxville, Knoxville, TN, United States; ^6^Department of Biochemistry & Cellular and Molecular Biology, The University of Tennessee, Knoxville, Knoxville, TN, United States

**Keywords:** proteomics, lipidomics, phospholipids, surfactant, swarming, flagella, exopolysaccharides, glycerol

## Abstract

*Paenibacillus polymyxa* is a Gram-positive bacterium commonly found associated with plant roots. *P. polymyxa* can exhibit two forms of flagellar motility: swimming in liquid culture and swarming on a surface. Here, swimming cells were compared to swarming cells using an integrated proteomic and lipidomic approach, yielding information about how lipid modifications and protein/enzyme pathways are tailored for these specific phenotypes. Observed differences in both phospholipid composition and metabolism between the two conditions suggest membrane remodeling in response to the surrounding environment. Key enzymes involved in glycerophospholipid metabolism were abundant in swimming bacteria, while enzymes associated with glycerol-3-phosphate metabolism were more abundant in swarming bacteria. Several glycoside hydrolases were either unique to or more abundant during swarming. This likely reflects the degradation of their own exopolysaccharides to both enhance swarming and supply the necessary chemical energy to compensate for increased flagellar synthesis. The observed upregulation of biosynthetic gene clusters (polyketides, lantibiotics, and surfactin) in swarming bacteria suggest the importance of signaling, antimicrobial activity, and surfactin production during this mode of motility – the latter of which is confirmed via RT-PCR.

## Introduction

Bacteria achieve motility through flagella assemblies that allow them to control directional movement, such as swimming in liquid cultures. However, microorganisms can also sense and respond to their surrounding environment, which can result in complex behaviors at the community-level. Swarming motility, a highly coordinated process in some bacterial populations to rapidly colonize a surface, obligately depends on cell-to-cell communication, and enables adaptation mainly driven by changes in the local environment (Copeland and Weibel, 2009). For example, swarming motility is important during root colonization (in the case of rhizobacteria) or for rapid colonization of a specific tissue (Gao et al., 2016). Biofilm development and swarming motility depend on both chemical sensing of the local environment, as well as mechanosensing of physical surfaces. For example, inhibition of flagellar rotational movement increases biofilm formation but negatively impacts swarming motility in *Bacillus subtilis* (Cairns et al., 2013).

Swarming and swimming motility are active forms of movement driven by flagella. While swimming motility is observed at an individual microbial level, swarming motility is driven by migration as multicellular groups (i.e., rafting) moving across a surface (Kearns, 2010). Microscopy studies of swarming *Proteus mirabilis* revealed extensive rafting and possible hyperflagellation (Jones et al., 2004). Hyperflagellation, a dramatic increase in flagellar density during swarming motility, has been observed as critical for increasing the total amount of power generated by flagella (Hall et al., 2017). It appears that a threshold amount of power is needed for an organism to swarm, and additional flagella contribute toward a cumulative increase in power. Besides multicellular rafts of highly flagellated cells, swarming motility also requires the secretion of lipopeptide surfactants/non-ribosomal peptides that act as wetting agents during bacterial growth on plates, thus providing a mechanism to help them spread rapidly over surfaces (Arima et al., 1968; Matsuyama et al., 1992; Lindum et al., 1998; Kearns and Losick, 2003; Leclère et al., 2006; Kearns, 2010; Mukherjee et al., 2015). The importance of non-ribosomal peptides to swarming motility has been previously demonstrated in *P. mirabilis*, whereby mutations to a gene encoding a non-ribosomal peptide/polyketide synthase lead to a swarming-deficient strain (Gaisser and Hughes, 1997). Additionally, active antimicrobial compounds have been reported to play a role in swarming motility; for example, bacilysocin probably derived from phospholipid phosphatidylglycerol through ester hydrolysis in *B. subtilis* has been found to be antimicrobial (Stein, 2005).

Bacteria can adjust their membrane lipid composition to maintain phospholipid homeostasis in order to adapt to different growth conditions/environmental perturbations, for example lysyl-phosphatidylglycerol (LPG) can protect *Listeria monocytogenes* from osmolytic stress and regulate the motility operon (Dare et al., 2014). Thus, in order to understand how membrane composition affects bacterial motility, it is critical to measure the range and changes in abundance for phospholipids associated with this swarming phenotype.

To unravel the underlying molecular details of swarming bacteria, this study focused on comparing and contrasting swimming vs. swarming growth conditions in *P. polymyxa* ATCC 842, which is known to exhibit a swarming phenotype (Park et al., 2008). *Paenibacillus* spp. are Gram-positive bacteria that are found in a range of soil, water, and rhizosphere environments. Many *Paenibacillus* spp. are known to be important endophytic, nitrogen-fixing rhizobacteria (Ding et al., 2005). For example, *P. polymyxa* is often employed as a plant growth-promoting rhizobacterium in horticulture and agriculture (Timmusk et al., 2005). Its use is primarily driven by its repertoire of biosynthetic genes for antibiotics and a number of extracellular hydrolytic enzymes (Kim et al., 2010; Jeong et al., 2011; Xie et al., 2015). In the *Paenibacillus* genus, the complex modular organization, including swarming, of *Paenibacillus vortex* has been examined, revealing how morphological changes were accompanied by high expression of some flagellar genes and resistance toward antibiotics (Roth et al., 2013).

Most swarming studies have been limited to microscopic-based morphological studies of bacterial cells, western blotting experiments, RT-PCR, construction of mutants, and motility assays (Kearns and Losick, 2003; Connelly et al., 2004; Kearns et al., 2004; Calvio et al., 2005; Julkowska et al., 2005; Kearns, 2010; Hall et al., 2017), but a detailed molecular-level interrogation of the phospholipids and protein metabolic pathways tailored to swarming motility has not been reported. Therefore, this study was designed to focus on a comprehensive integrated proteomic and lipidomic measurement approach to highlight a detailed view of the molecular machinery linked to this interesting phenotype. This study generated composite omics datasets, which revealed growth condition-dependent phospholipid metabolism, particularly from phosphatidic acid (PA) and sn-glycerol-3-phosphate, and subsequent membrane remodeling. This integrated approach also identified a possible pathway to generate energy that may be required for swarming phenotype. Additionally, this study examined potential roles of glycoside hydrolases, flagellar assembly, chemotaxis, wetting agents, and surfactants in swarming motility.

## Materials and Methods

### Bacterial Strains and Growth Conditions

**FIGURE 5:**
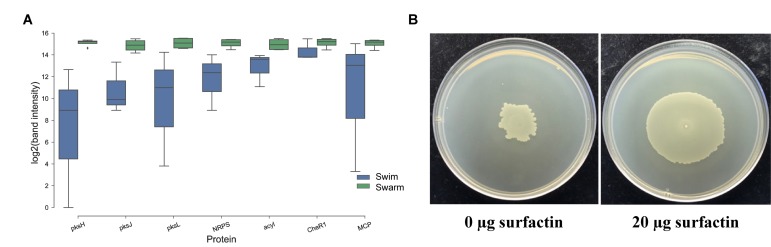
Follow up experiments to further characterize the expression of key, highly abundant proteins and surfactin’s role in swarming. **(A)** RT-PCR was performed on normalized RNA concentration extracted from *P. polymyxa* growing in liquid media (Swim) and the swarm edge (Swarm). Most of the genes that belong to PKS were seen upregulated in swarming compared to swimming bacteria. Some chemotaxis related genes (CheR1 and MCP) were seen upregulated in swarming bacteria. Surfactin (srfAA_4), significant with proteomics experiment, was also significantly upregulated in swarming bacteria when compared to swimming. **(B)** An exogenous addition of commercial surfactin (20 μg) dramatically enhances swarming motility in *P. polymyxa*. The figure is taken after bacteria were allowed to swarm in Muller-Hinton Agar (1.7% agar) for 2 days.

*Paenibacillus polymyxa* ATCC842 was obtained from The American Type Culture Collection (Manassas, VA, United States). Swarming agar plates were made using Müller-Hinton media (Sigma-Aldrich) with 1.7% agar. Samples for experiments involving swimming bacteria were collected by growing cells overnight in Müller-Hinton broth with shaking at 30°C to a low optical density (OD600 < 0.6). The cells were then collected by centrifugation (5000 rpm, 10 min) and washed once in sterile PBS prior to analyses. Studies using cells grown under swarming conditions were performed by inoculating the center of a Müller-Hinton swarming agar plate with 5 μL of liquid culture and incubating at 28°C for 4–5 days to allow for sufficient swarming and enabling us to separate out cells that were actively growing and swarming by collecting cells from the outer 0.5 cm of the swarm edge. Cells were collected using a sterile loop to scrape around the edge of the swarm and placing the collected cells in sterile PBS, then washing once before pelleting the cells as described above and stored at −80°C for further analyses. Viability of cells collected from the outer edge of swarms was tested using a LIVE/DEAD BacLight Bacterial Viability Kit (Thermo Fisher Scientific) and >90% of cells were found to be still viable. The effects of surfactants on swarming were tested by adding 10 μl of surfactin (Sigma) at specific concentrations (Figure 5) to the center of swarming agar and allowing the plates to dry overnight before inoculating as described above.

### Proteome Characterization Using LC-MS/MS

Washed cell pellets (triplicates) per condition (∼50 mg each) were resuspended in lysis buffer (250 μL of 4% sodium deoxycholate (SDC) in 100 mM ammonium bicarbonate (ABC) pH 8.0) and boiled at 95°C for 5 min. Samples were further lysed via sonic disruption (Branson Sonifier), and centrifuged to remove cell debris, diluted with 250 μL of ABC to bring the SDC concentration to 2%, and filtered using 10 kDa MWCO spin column (Vivaspin500; Sartorius). Concentrated proteins were then washed with 500 μL of ABC on top of the filter (SDC concentration is now adjusted to ∼1%). The resolubilized proteins were transferred to an eppendorf tube and concentrations were assessed by BCA (Pierce). Proteins were reduced with 5 mM DTT, incubated in 37°C for 30 min, alkylated with 15 mM IAA, stored in dark for 30 min at room temperature to block disulfide bridge reformation, and digested to peptides with two sequential aliquots of sequencing-grade trypsin (Promega Corp., Madison, WI, United States) at a 1:50 enzyme:protein ratio (w/w), initially overnight then followed by 4 h at room temperature. Formic acid (0.5%) was added and samples were centrifuged (21,000 × *g*) to precipitate SDC and collect tryptic peptides. The tryptic solution was transferred to a new tube and a BCA protein assay was done to estimate the amount of tryptic peptides per sample. A 25-μg aliquot of peptides was pressure loaded onto a biphasic back column [∼4 cm strong cation exchange (SCX) followed by ∼4 cm reverse-phase (C18) material]. Bound peptides were then washed offline with solvent A [95% HPLC grade water, 5% acetonitrile, 0.1% formic acid (FA)] for 20 min, followed by a 25 min gradient to solvent B (70% acetonitrile, 30% HPLC grade water, 0.1% FA). Next, back-column was coupled in-line with an in-house pulled, reverse-phase (∼15 cm) packed nanospray emitter and analyzed via 22-h MudPIT (multidimensional protein identification technology), as described previously (Washburn et al., 2001; Mcdonald et al., 2002). Peptides were separated in 11 steps (each lasting ∼2 h) with an increasing amount of ammonium acetate (25, 30, 35, 40, 45, 50, 65, 80, 100, 175, and 500 mM) followed by organic gradients in each step. Peptide sequencing analysis was performed with an LTQ-Orbitrap-Velos-Pro mass spectrometer (Thermo Fisher Scientific, San Jose, CA, United States) in a data-dependent mode with each full scan (1 microscan) collected at 30,000 resolution in Orbitrap mass analyzer, followed by CID fragmentation of the 20 most abundant ions (1 microscan). Dynamic exclusion was enabled with a mass exclusion width 20 ppm and exclusion duration 30 s.

MS/MS data were searched against the *P. polymyxa* ATCC 842 proteome (Assembly GCA_000217775.1), concatenated with common contaminant proteins (e.g., trypsin and human keratin), and reversed sequences of the target database using Tide-search (Diament and Noble, 2011) using following parameters: parent mass tolerance of 30 ppm, a static modification on cysteine (+57.0214 Da), and a dynamic modification to an oxidation (+15.9949 Da) of methionine (rest parameters were assigned as default), followed by Percolator (Käll et al., 2007) with a test-FDR and train- FDR of 0.05, along with rest default parameters to assign spectra to peptides (peptide-spectrum matches; PSM). Retention times of each PSM were extracted parsing mzML file with in-house script and MS1 apex intensities were assigned using moFF (Argentini et al., 2016). The moFF parameters were set to 30 ppm for the precursor mass tolerance, 5 min for the XIC time window, and 1 min (equivalent to 60 s) to get the apex for the ms2 peptide/feature. The peptide intensities from each salt pulse were summed to their respective proteins per sample. Protein intensities were then normalized by protein length and overall abundance per MS run. Each protein identification required a minimum of 2 distinct peptides and 2 PSMs. For quantitative comparisons, only proteins identified in 2 out of 3 replicates were considered. The resulting matrix of protein abundance values was log2-transformed and missing values imputed such that the mock value falls at 2.8 standard deviations from the mean of the log-normal distribution of protein abundances. To obtain a better depth in annotations of the identified proteins, eggNOG mapper (Huerta-Cepas et al., 2017), BlastKOALA (Kanehisa et al., 2016) and Prokka (Seemann, 2014) were used. Student’s *t*-test was performed to obtain the differentially abundant proteins across conditions (*p*-value < 0.05, absolute value of Log2 fold-change >1). Thus obtained differentially higher abundant proteins for each conditions (swarming and swimming) were assessed for Gene Ontology (GO) enrichment using ClueGO (Bindea et al., 2009). The GO terms were subjected to the right-sided hypergeometric enrichment test and *p*-value correction was performed using Benjamini–Hochberg method. The GO terms at adjusted *p* < 0.05 were considered significantly enriched. Also, KEGG map (Kanehisa and Goto, 2000) obtained from eggNOG mapper were evaluated for functional enrichment (swarming and swimming) using Fisher’s exact test through PIANO [25] package and KEGG map with *p* < 0.05 were considered significantly enriched. The KEGG Orthology and Enzyme Commission (EC) numbers obtained from BlastKOALA and Prokka served as the basis for investigating different metabolic pathways. Homology searches of proteins from known metabolic pathways in *B. subtilis* and other *Paenibacillus* spp. were performed using BlastP. All raw mass spectra for the proteome measurements have been deposited into the ProteomeXchange repository with the following accession numbers: (MassIVE Accession: MSV000083145, ProteomeXchange: PXD011747, FTP link to files: ftp://MSV000083145@massive.ucsd.edu, Reviewer password: “abcd1234”).

### Lipidome Characterization Using LC-MS/MS

Lipids were extracted using a modified version of the protocol as published previously (Guan et al., 2010). Cell pellets were resuspended in 1 mL of 95% EtOH, water, diethyl ether, pyridine, and 4.2 N ammonium hydroxide 15:15:5:1:0.18. Then, 100 μL of glass beads were added and the sample was vortexed. The samples were incubated at 60°C in a water bath for 20 min and then centrifuged at 10,000 × *g* for 10 min. The supernatant was removed and added to a vial to dry. This extraction was repeated and the supernatant was added to the same vial for drying. Then 300 μL of water saturated butanol and 150 μL of water was added to the Eppendorf tube, vortexed, and centrifuged at 10,000 × *g* for 2 min. The top butanol phase was placed in the same vial for drying. The aqueous phase was re-extracted with 300 μL of water saturated butanol, vortexed, and centrifuged at 10,000 × *g* for 2 min and the top butanol phase was added to the same vial for drying. Lipid extracts were dried under N_2_, resuspended in 300 μL of MeOH:CHCl_3_ (9:1) and placed in an autosampler vial for MS analysis.

An Ultimate 3000 autosampler and UPLC pump (Thermo Fisher Scientific, San Jose, CA, United States) were used to separate extracted lipids on a Kinetex HILIC column (150 mm × 2.1 mm, 2.6 μm) (Phenomenex, Torrance, CA, United States). Analytes were then introduced to an Exactive benchtop Orbitrap mass spectrometer (Thermo Fisher Scientific, San Jose, CA, United States) via an electrospray ionization (ESI) probe. The total run time for each analysis was 35 min with mobile phase A and B consisting of 10 mM aqueous ammonium formate adjusted to pH 3 in 93% (v/v) ACN and 10 mM ammonium formate adjusted to pH 3, respectively. A flow rate of 0.2 mL/min was used with the gradient as follows: *t* = 0 min 100% A, *t* = 15 min 81%A, *t* = 15.1 min 48% A, *t* = 25 min 48% A, *t* = 25.1 min 100% A, *t* = 35 min 100% A. The temperature of the column oven was 25°C and the temperature of the autosampler was 4°C.

All samples were analyzed in positive and negative mode with a resolution of 140,000 k using a scan range of 100–1500 *m/z*. For both positive and negative mode analyses the ESI source settings were the same. For all analyses, the heated capillary was set to 350°C, the spray voltage was 4 kV, the sheath gas flow was set to 25 units, and the auxiliary gas set to 10 units. The standard calibration protocol from Thermo Fisher was performed approximately every 48 h for external mass calibration.

Maven software was used to integrate the chromatographic peaks (Melamud et al., 2010). Lipids were identified using both the exact *m/z* of the parent ion and their respective retention times. Concentrations were calculated by external calibration curves for lipid standards (Avanti Polar Lipids, Alabaster, AL, United States) from each of the following lipid groups: sulfoquinosyldiacylglycerol (SQDG), monogalacto diacylglycerol (MGDG), phosphatidylserine (PS), phosphatidylglycerol (PG), phosphatidylethanolamine (PE), PA, cardiolipin (CL), ceramide (Cer), lysophosphatidic acid (lysoPA), and lysophosphatidylethanolamine (lysoPE). The following molecule groups were identified based on the exact m/z of the parent ions: sn-glycerol 3-phosphate (sn-G3P), sn-glycero-3-phosphoethanolamine (sn- G3PE), sphingomyelin (SM), sulfatide (GalβCer), cytidine diphosphate diacylglycerol (CDP-DAG), lactosylceramide (LacCer), glucosylceramide (GlcCer), sn-glycero-3-phosphocholine (G3PC) and acetylcholine (AC). The intensity (measured as ion counts) for each metabolite was normalized to cell number in order to allow comparison of relative concentrations (ion counts/cell) among different samples. Student’s *t*-test was performed to obtain the differentially abundant phospholipids across conditions (*p*-value < 0.05, absolute value of Log2 fold-change >1).

### Confirming Gene Expression With RT-PCR

*Paenibacillus polymyxa* was grown on Müller-Hinton media in either liquid media (swimming) or on semi-solid agar (swarming) as described above prior to extraction of RNA with the Qiagen RNeasy Mini Kit according to manufacturer’s protocols. RNA concentrations were normalized using a NanoDrop and cDNA was generated using the ThermoScript RT-PCR System from Thermo Fisher Scientific according to the manufacturer’s protocols. Primers for PCR were designed to amplify an approximately 500 base pair region from each gene of interest [PksH (WP_019687993.1), PksJ (WP_019687990.1), PksL (WP_019687991.1), NRPS (WP_019687302.1), Acyltransferase (WP_016822428.1), CheR (WP_017425950.1), MCP (WP_019688156.1)] and PCR was performed on cDNA using the FailSafe PCR System from Lucigen based on manufacturer’s recommendations.

## Results and Discussion

### Swarming *P. polymyxa* Reveal Significant Phospholipid Modification

**FIGURE 1:**
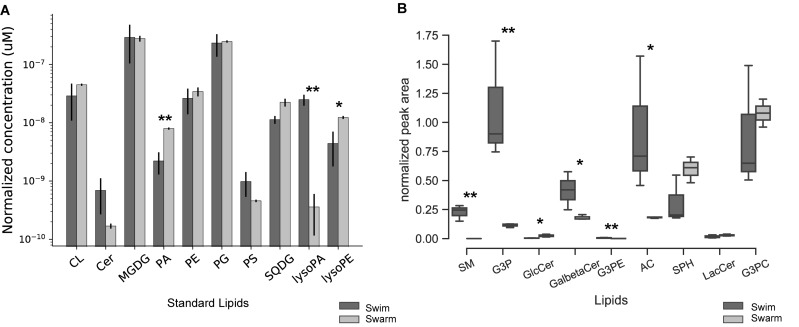
Quantification of phospholipids. **(A)** Bar graph of the phospholipids quantified from swarming and swimming *P. polymyxa*. The *y*-axis is the amount of normalized phospholipid concentration and *x*-axis is the ranges of quantified phospholipids. The black bar represents the swimming motility and gray bar represents swarming motility. The samples were normalized by cell counts (CL, cardiolipin; Cer, ceramide; MGDG, monogalacto diacylglycerol; PA, 1,2-diacyl-sn-glycerol-3P (phosphatidic acid); PE, phosphatidylethanolamine; PG, phosphatidylglycerol; PS, phosphatidylserine; SQDG, sulfoquinovosyl diacylglycerol; lysoPA, lysophosphatidic acid; lysoPE, lysophosphatidylethanolamine). Swim – swimming motility, Swarm – swarming motility. **(B)** Boxplots showing the normalized peak areas of phospholipids for swimming and swarming culture. The samples were normalized by cell counts. (SM, sphingomyelin; G3P, sn-glycerol-3-phophate; GlcCer, glucosylceramide/galactosylceramide; GalbetaCer, sulfatide; G3PE, sn-glycero-3-phosphoethanolamine; AC, acetylcholine; SPH, sphingosine; LacCer, lactosylceramide/digalactosylceramide; G3PC, sn-glycero-3-phosphocholine). Swim – swimming motility, Swarm – swarming motility (^∗^ *p* < 0.05, ^∗∗^ *p* < 0.01).

In response to environmental changes, bacteria have the ability to alter the fluidity of their membrane by changing their lipid composition. The lipid bilayer contains phospholipid head groups with attached acyl chains that usually contain 12–22 carbons, with or without double bonds (Milne et al., 2006). The combinations of head groups and acyl chain structures determine the physiochemical properties of the membrane, and bacteria can redistribute acyl chains and phospholipid moieties in response to their surroundings (Zhang and Rock, 2008). Lipids in *Bacillus* and *Paenibacillus* contain predominantly straight-chain and branched-chain fatty acids (Kämpfer et al., 2012). For *P. polymyxa*, lipidomic results indicate that there are clear differences observed across lipids groups when comparing swarming bacteria to swimming bacteria (Figure 1A). Four lipid groups (PA, lysoPA, SQDG, and lysoPE) were significantly altered, but the remaining groups of lipids (CL, Cer, MGDG, PE, PG, and PS) were unchanged. Among the four lipid groups that significantly differed, PA, SQDG, and lysoPE were at higher abundances in swarming bacteria, while lysoPA was more abundant in swimming bacteria. Details of the quantified lipids are summarized in Supplementary Table S1. In addition to these lipids, other intermediates (sn-G3P, sn-G3PE, SPH, SM, GalβCer, sulfatide, CDP-DAG, LacCer, and GlcCer lipids) of glycerophospholipid and glycolipid metabolism were identified, and their relative abundances were measured. Normalized peak areas for these lipids are illustrated as boxplots in Figure 1B. sn-G3P, SM, GalβCer, G3PE, and AC were significantly higher in swimming conditions as compared to swarming.

**FIGURE 2:**
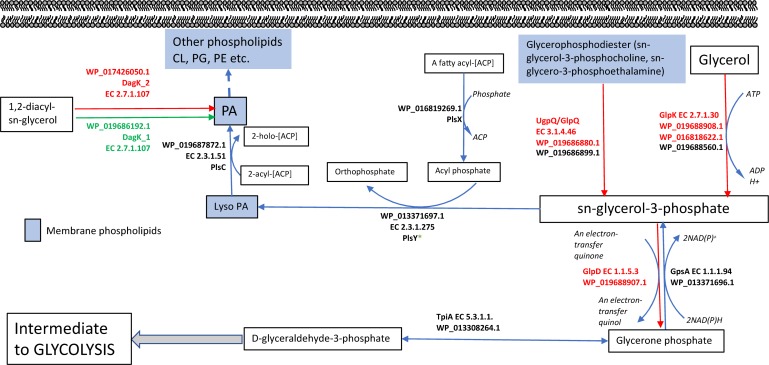
Metabolic fate of sn-glycerol-3-phosphate (sn-G3P): All the enzymes shown were measured by LC-MS/MS. sn-G3P (central molecule) is the precursor for synthesis of glycerophospholipids. 1,2 diacyl-sn-glycerol can also form lysoPA that ultimately forms PA. Two DagK enzymes responsible for forming lysoPA are significantly upregulated with several folds in swimming and swarming condition. sn-G3P can also be formed from glycerophosphodiester and proteins responsible for this step are specific for swarming activity suggesting a constant and active turnaround of sn-G3P from phosphodiester. sn-G3P is also a key intermediate of glycolysis. The red-colored enzymes and arrows are significantly higher in abundance in swarming motility, green-colored enzymes and arrows are significantly highly abundant in swimming motility and black color enzymes indicate that these enzymes were detected using MS but no significant difference in protein abundance in both motilities.

Collectively, these data reveal differences in the phospholipid composition between swimming and swarming bacteria. Some of these differentially abundant phospholipid groups are known to play crucial roles in adapting cells to their environment. PA serves as the universal precursor and central intermediate in the formation of membrane glycerolipids (Zhang and Rock, 2008). A pathway for the biosynthesis of lipids in *P. polymyxa* that highlights differentially abundant enzymes measured by proteomics is shown in Figure 2. sn-G3P is one of the most important precursors for phospholipid biosynthesis (Morbidoni et al., 1995), and a lower abundance of sn-G3P in swarming bacteria might indicate a continuous utilization during swarming. PlsX (WP_016819269.1), which is responsible for production of acyl phosphate, is the key step in sequential transfer of acyl-phosphate (acyl-PO_4_) to sn-G3P to form LysoPA by PlsY (WP_013371697.1). PlsX was detected as highly abundant in both growth conditions, whereas, PlsY was measured only in swimming bacteria, but at low abundance, supporting our observation of LysoPA accumulation in swimming bacteria compared to swarming bacteria (Figure 1A). While PlsC (WP_019687872.1) can convert LysoPA to PA, diacylglycerol kinase (DagK) can also convert 1,2-diacyl-sn-glycerol to PA. In *P. polymyxa*, two homologs of DagK were detected; DagK1 (WP_019686192.1) was more abundant in swimming bacteria (∼6×) while DagK2 (WP_017426050.1) was measured only in swarming bacteria, ∼18× higher in abundance when compared to swimming after data imputation. Figure 2 reveals the conversion of 1,2-diacyl-sn-glycerol to PA, facilitated by diacylglycerol kinase. The presence of DagK2 only in swarming bacteria in addition to DagK1 also corroborates the accumulation of PA in swarming bacteria (Figure 1A).

Previous studies have shown how the biochemical properties of PA and LysoPA can affect local membrane curvature in that PA has negative spontaneous curvature while LysoPA has positive spontaneous curvature (Kooijman et al., 2003, 2005). Additionally, PA has been reported to regulate intracellular membrane transport, alter biophysical properties of the membrane, and induce membrane bending and destabilization (Kooijman et al., 2003). Based on our measurements, accumulation of PA in swarming bacteria and LysoPA in swimming bacteria suggests membrane remodeling as a function of phenotype. A recent study on Gram-negative cells of *Pseudomonas aeruginosa* highlighted a newly identified PA binding protein (PA3911) that had significant influence on the lipid homeostasis and related macroscopic phenotypes (Groenewold et al., 2018). However, since *P. polymyxa* is a Gram-positive bacterium with a differing phospholipid composition and since a homolog of PA3911 was not found (Blastp analysis) in *P. polymyxa* ATCC 842, the evidence of PA as the signaling molecule for *P. polymyxa* ATCC 842 seems less likely than lipid remodeling.

In conjunction with the lipidomics measurements (Figure 1A), the proteomic data support the observed metabolism of ceramide in swarming bacteria. The reference strain of *P. polymyxa* SC2 consists of three proteins annotated to be involved in ceramide metabolism. Corresponding ceramide reactions were evaluated in this study, where LacZ1 (and/or other beta-galactosidases) and MelA (alpha-galactosidase) can produce GlcCer from LacCer with the release of a galactose molecule as shown and described in Supplementary Figure S1. WP_019685818.1 is 98% identical to PPSC2_01420 and is annotated as a beta-galactosidase (LacZ1), which is known to hydrolyze terminal non-reducing β-D-galactose residues in β-D-galactosides. This protein (LacZ1) was ∼8× more abundant in swarming bacteria. Another homolog of LacZ1 (WP_019686095.1) was also more abundant (10×) in swarming bacteria. LacZ1 is known for conversion of lactosylceramide to glucosylceramide, with a release of galactose. Similarly, WP_019686312.1 is annotated as MelA [an alpha-galactosidase (EC 3.2.1.22)], and is involved in conversion of digalactosylceramide to galactosylceramide with a release of galactose. In swarming, WP_019686312.1 (MelA) is capable of hydrolyzing terminal non-reducing alpha-D-galactose residues in alpha-D-galactosides and was detected ∼70× more abundant in swarming bacteria. This enzyme (EC 3.2.1.22 and K07406) is associated with several KEGG pathways (ko00052-Galactose metabolism, ko00561-Glycerolipid metabolism, ko00600-Sphingolipid metabolism, ko00603-Glycosphingolipid biosynthesis – globo and isoglobo series) and is capable of 12 different enzymatic reactions (Kanehisa et al., 2011). The measurement of these enzymes found in the ceramide metabolism pathway suggests that sphingolipids could play a role in galactose metabolism. Thus, the possible role of galactose metabolism in swarming bacteria is discussed below. In total, the lipidomics data revealed significant alterations of key phospholipids in the swarming phenotype, suggesting a substantial remodeling of the cellular membrane in this phenotype. In particular, the accumulation of PA in swarming *P. polymyxa* suggests an alteration of the biophysical properties of the membrane.

### Swarming *P. polymyxa* Reveal Elevated Abundances of Enzymes Involved in Carboxylic and Pentose Catabolic Processes

To characterize the range of proteins vital for defining phenotypes in *P. polymyxa* ATCC 842, we sought to comprehensively measure the expressed proteomes under the two distinct phenotypic conditions. A total of 39,179 peptides mapping to 3,072 proteins were quantified across both conditions. Principle component analysis (Supplementary Figure S2) highlights the discrete grouping of biological replicates under both growth conditions. In total, 2,629 proteins (86%) were identified in both conditions. Even though a high percentage of overlap was observed, 233 proteins (7.5%) were only identified in swarming bacteria, while 210 proteins (6.8%) were identified only in swimming bacteria.

**FIGURE 3:**
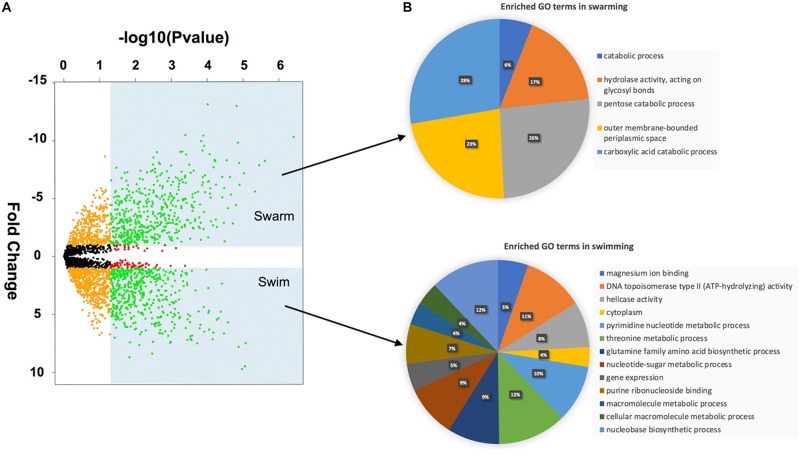
Volcano plot and GO enrichment of differentially abundant proteins: **(A)** Volcano plot -Green dots in the shaded regions are the proteins that have a *p*-value < 0.05 and FC > 1× (log scale). **(B)** Gene Ontology enrichment using ClueGO.

Student’s *t*-test revealed that 528 proteins were significantly more abundant in swarming, while 566 proteins were significantly more abundant in swimming bacteria (Supplementary Table S2). To highlight the biggest changes, the *p*-values and fold changes were plotted in a volcano plot (Figure 3A). Both of the groups represented by green dots were investigated separately for their GO terms and their KEGG enrichment. The GO enrichment results reveal several key differences between swarming and swimming motility (Figure 3). Figure 3 shows a pie-chart constructed using percentages of associated proteins for individual GO terms. In total, 31 proteins with GO terms were found to be functionally enriched in swarming bacteria in the five broad categories, as shown in Figure 3B (upper figure). In swimming bacteria, a total of 58 GO terms were enriched, of which 13 representative GO terms are shown in Figure 3B (lower figure). The detailed functions of these proteins are listed in Supplementary Table S3. Some of these GO terms are described below.

One of the enriched GO terms in swarming phenotype was pentose catabolic processes, which includes xylose isomerase (WP_016820313.1), L-ribulose-5-phosphate 4-epimerase (WP_016822497.1) and L-arabinose isomerase (WP_016822498.1). All of these enzymes are involved in the production of D-xylulose/D-xylulose-5-phosphate. Additionally, transaldolase (WP_013370592.1) was significantly highly abundant in swarming bacteria, indicating a strong need for the pentose phosphate pathway during swarming motility.

Similarly, proteins enriched with carboxylic acid catabolic process were also abundant in the swarming phenotype. Within this GO term, WP_019688294.1 is annotated as aminomethyltransferase (GcvT), which is known to catalyze the degradation of glycine via the glycine cleavage system. Along with GcvT, three other proteins of glycine cleavage system GcvH (WP_016819056.1), GcvPA (WP_019688295.1) and GcvPB (WP_019688296.1) were significantly more abundant or only detected in swarming bacteria. A previous study on *B. subtilis* revealed the importance of the *gcvT* operon in swarming motility (Babina et al., 2017). Likewise, the glycine cleavage system appears to be crucial to *P. polymyxa* swarming motility, as the entire operon was highly abundant. Similarly, enrichment of KEGG maps, as shown in Table 1, also revealed enriched catabolic processes involving carboxylic acid, pentose sugars and small molecules, 5-carbon sugar metabolism, fructose and mannose metabolism, TCA cycle, pathways involving chemotaxis, and galactose metabolism.

**TABLE 1:** KEGG pathway enrichment in swarming bacteria and swimming bacteria.

**KEGG pathway**	***p*-value (BH adjusted) <0.05**
**Pathways enriched in swarming bacteria**	
map01100_Metabolic pathways	1.46E-09
map02030_Bacterial chemotaxis	4.49E-05
map00030_Pentose phosphate pathway	0.019011534
map01110_Biosynthesis of secondary metabolites	1.61E-05
map01120_Microbial metabolism in diverse environments	2.12E-05
map00051_Fructose and mannose metabolism	0.007826065
map00040_Pentose and glucuronate interconversions	4.19E-07
map02020_Two-component system	0.031028479
map00363_Bisphenol degradation	0.026683197
map00625_Chloroalkane and chloroalkene degradation	0.025922718
map00500_Starch and sucrose metabolism	0.00185018
map00020_Citrate cycle (TCA cycle)	0.002481061
map00720_Carbon fixation pathways in prokaryotes	0.026683197
map04626_Plant-pathogen interaction	0.026683197
map00052_Galactose metabolism	0.017939536
map00730_Thiamine metabolism	0.021470413
map00740_Riboflavin metabolism	0.026683197
map00380_Tryptophan metabolism	0.021470413
map04973_Carbohydrate digestion and absorption	0.008902383
**Pathways enriched in swimming bacteria**	
map03010_Ribosome	0.040614334
map01100_Metabolic pathways	8.51E-06
map01110_Biosynthesis of secondary metabolites	0.022710687
map00240_Pyrimidine metabolism	0.004298894
map00450_Selenocompound metabolism	0.014127524
map00250_Alanine, aspartate and glutamate metabolism	0.022710687
map05120_Epithelial cell signaling in *Helicobacter pylori* infection	0.029663556

The GO enrichment results for swimming bacteria revealed cytoplasmic biosynthesis pathways including ribosome biosynthesis, DNA replication machinery, and many more as listed in Supplementary Table S3. Similarly, KEGG enrichment revealed ribosome related pathways, pyrimidine metabolism, and seleno-compound metabolism.

Overall, the GO enrichments and KEGG pathway enrichments revealed that swarming motility in *P. polymyxa* depends on enzymes involved in carboxylic and pentose catabolic processes such as glycine cleavage pathways and hydrolase activity (as discussed below). Besides flagellar biosynthesis, catabolic processes seems critical to swarming *P. polymyxa*, as suggested by the ribose transport system, and pentose phosphate pathways. The elevated abundance of transaldolase (WP_013370592.1) in swarming correlates well with the conversion of D-glyceraldehyde-3-phosphate to fructose-6-phosphate to enter glycolysis. Some of the key enrichment pathways revealed in Figure 3 were examined in more detail, as highlighted in the sections below. The possible pathway for generation of D-glyceraldehyde-3-phosphate in swarming bacteria is also explained below.

### Swarming *P. polymyxa* Appear to Regulate Glycerol for Enhanced Energy Demands

sn-G3P is known to be the key precursor for biosynthesis of phospholipids. There is continuous replenishment of sn-G3P via degradation of phospholipids. As shown in Figure 2, there are multiple fates of sn-G3P. In *B. subtilis*, the *glpPFKD* gene cluster is crucial to the organism’s growth on glycerol or sn-G3P (Beijer et al., 1993). Although not much is known about *P. polymyxa* glycerol metabolism, the integration of lipidome and proteome data in this study provided some valuable insights into the regulation of glycerol in swarming bacteria as compared to swimming bacteria. sn-G3P (Figure 1B) was significantly more abundant in swimming bacteria when compared to swarming bacteria, suggesting its accumulation rather than utilization. Swarming bacteria need more ATP for flagellar activity, so glycerol likely serves as an intermediate for energy production. sn-G3P in *P. polymyxa* can be formed via glycerone phosphate or could result from the phosphorylation of glycerol and proceed to form glycerone phosphate. Enzymes capable of doing either of these processes were detected by LC-MS/MS. In swarming bacteria, the latter process seems more relevant, as several glucose starvation proteins are highly abundant, and this process would eliminate the need to utilize glucose. Three homologs (WP_019688908.1, WP_016818622.1 and WP_019688560.1) of glycerol kinase (EC 2.7.1.30), GlpK, were detected in this study. As shown in Figure 2, two of these homologs (WP_019688908.1 and WP_016818622.1) are significantly more abundant in swarming bacteria, by 6× and 13×, respectively.

Glycerophosphodiester can be hydrolyzed to sn-G3P by glycerophosphoryl diester phosphodiesterase (EC:3.1.4.46), GlpQ. It can release sn-G3P from a broad range of substrates: glycerophosphoethanolamine, glycerophosphocholine, glycerophosphoglycerol, and bis (glycerophospho) (Larson et al., 1983). GlpQ (WP_019685880.1) was significantly more abundant (6×) in swarming bacteria. This suggests continuous breakdown of glycerophosphodiester in swarming bacteria, which is actively converted to sn-G3P (a precursor to lipid formation). sn-G3P dehydrogenase (GlpD) is capable of converting sn-G3P to glycerone phosphate (dihydroxyacetone phosphate), an intermediate of glycolysis. GlpD (WP_019688907.1) was significantly more abundant during swarming (6×). In fact, proteomics results show that the entire GlpPKD operon (WP_019688907.1: GlpD, WP_019688908.1:GlpK_3 and WP_019688909.1:GlpP) was significantly upregulated in swarming bacteria, suggesting increased energy demand and utilization. A reversible enzyme triose phosphate isomerase, TpiA (WP_013308264.1), measured as highly abundant in both forms of motility, converts glycerone phosphate to D-glyceraldehyde-3-phosphate, an intermediate for glycolysis. As mentioned earlier, it also can feed into the pentose phosphate pathway to generate fructose-6-phosphate (also another intermediate of glycolysis). The presence of GlpD at higher abundance in swarming explains the conversion of sn-G3P to glycerone. The increased abundance of proteins involved in the TCA cycle (Table 1) is consistent with the generation of additional energy during swarming motility.

### Increased Abundance of Glycoside Hydrolases (GH) Suggest a Way to Supply Nutrient and Energy Demands in Swarming *P. polymyxa*

The draft genome of *P. polymyxa* ATCC 842 was evaluated with eggNOG mapper, BlastKOALA, and Prokka in order to better annotate protein functional domains, EC numbers, and KO terms, as tabulated in Supplementary Table S4. Based on GO-term functional enrichment, proteins associated with hydrolase activity were further examined. A total of 15 proteins detected by proteomics were annotated as different families of GHs (GH4, 5, 16, 28, 31, 43, and 48). Among these, 10 were significantly more abundant (20–1200× increase) in swarming bacteria as compared to swimming (Table 2). In fact, 7 of these GHs were detected only in swarming bacteria. These proteins are capable of hydrolyzing a range of polysaccharides. For example, GH5 (WP_019688246.1), also known as cellulase (EC 3.2.1.4), is responsible for endohydrolysis of (1→4)-β-D-glucosidic linkages in cellulose, lichenin, and cereal β-D-glucans (Park et al., 2010). It was detected only under swarming conditions, suggesting a possible role of GHs in swarming motility. Another highly abundant enzyme, GH16 (WP_017426919.1) is known as beta-glucanase (EC 3.2.1.73), and catalyzes the hydrolysis of (1→4)-β-D-glucosidic linkages in β-D-glucans containing (1→3)- and (1→4)-bonds (Barras et al., 1969). CelY (WP_019687208.1), GH48, was ∼100× more abundant in swarming bacteria, and is responsible for the hydrolysis of (1→4)-β-D-glucosidic linkages in cellulose and cellotetraose, releasing cellobiose from the reducing ends of the chains (exoglucanase) (Berger et al., 2007).

*Paenibacillus polymyxa* is known to produce exopolysaccharide (EPS) (Haggag, 2007; Lal and Tabacchioni, 2009; Liu et al., 2009, 2010), which is comprised of various sugars such as glucose, galactose, mannose, and xylose (Haggag, 2007). Production of large amounts of EPS is a hallmark of biofilm formation in *P. polymyxa* (Haggag, 2007; Yegorenkova et al., 2011, 2013) and other microbes (Davies et al., 1993; Danese et al., 2000; Koo et al., 2010). The presence of exopolysaccharide would indicate a sessile lifestyle, and thus it should be absent (or minimal) in the swarming motility (Merritt et al., 2007). WP_019687364.1 has been annotated as exopolysaccharide biosynthesis protein and was significantly more abundant (∼44×) in swimming bacteria when compared to swarming bacteria. Since biofilm formation and swarming are inversely related (Verstraeten et al., 2008), this microbe may need to hydrolyze any EPS to enhance the swarming phenotype. In fact, GHs have been utilized to disrupt *P. aeruginosa* biofilms (Baker et al., 2016) and preformed fungal biofilms in order to reduce their virulence (Snarr et al., 2017). Therefore, the GHs detected in this study might be key players for hydrolyzing a broad range of these EPS, such as β-D-glucans. This further enhances the availability of nutrients which are crucial to sustain energy-demanding mechanisms, such as the metabolic cost associated with increased flagellar synthesis (Harshey, 2003).

It has been previously shown that deletion mutations of *P. polymyxa* E681 α-amylase and β-amylase genes PPE_02348 and PPE_04705 lead to full inhibition of starch degradation on a plate assay, confirming that both genes are required for starch utilization in E681 strain (Kim and Timmusk, 2013). Using eggNOG orthology search, WP_019687231.1 was detected as the ortholog of PPE_02348. Furthermore, a BlastP homology search of PPE_04705 revealed 40% identity with WP_019687823.1. Both of these proteins were present only in swarming bacteria and may be important in breakdown of starch to fulfill energy demands required for swarming motility in *P. polymyxa* ATCC 842. Other hydrolases that were highly abundant in swarming bacteria are reported in Table 2 along with their activities, i.e., chitinase, xylosidase, and arabinose.

**TABLE 2:** Glycoside hydrolases (GHs) specific to swarming motility; highly abundant and significant.

**Locus_tag**	**FC (Swarm-Swim) log2**	**Prokka Gene**	**Prokka EC**	**Prokka_annotation**	**HMM_model Annotation**	**KO**
WP_019688246.1	10.28		3.2.1.4	Endoglucanase E1	GH family 5	K01179
WP_017426919.1	9.02		3.2.1.73	Beta-glucanase	GH family 16	NA
WP_019687208.1	6.7	celY	3.2.1.91	Exoglucanase-2	GH family 48	NA
WP_019688013.1	6.5	pgl_3	3.2.1.15	Polygalacturonase	GH family 28	NA
WP_019688666.1	6.47	chiA1	3.2.1.14	Chitinase A1	GH family 81	NA
WP_019686312.1	6.1	melA_2	3.2.1.22	Alpha-galactosidase	GH family 4	K07406
WP_019688934.1	5.59		NA	NA	GH family 43	K06113
WP_019685857.1	5.34	abn2_1	3.2.1.99	Extracellular endo-alpha-(1→5)-L-arabinanase 2	GH family 43	K06113
WP_019686294.1	4.63	hypBA2	3.2.1.187	Beta-L-arabinobiosidase	GH family 5	NA
WP_019686956.1	4.61	yicI	3.2.1.177	Alpha-xylosidase	GH family 31	K01811

### A Key Characteristic of Swarming *P. polymyxa* Is Increased Abundance and Specialization of Flagellar Proteins

Although a hallmark of any type of cellular motility is related to flagellar activity, a major focus of this work was to systematically investigate whether there were key differences in flagellar production and signatures in swarming vs. swimming phenotypes. It is known that one of the most important requirements for swarming motility is flagellar biosynthesis (Kearns, 2010). As expected, several proteins associated with flagellar assembly were more abundant in swarming conditions, as compared to cells grown in liquid culture. In total, 35 detected proteins were linked to flagellation (Table 3); among them, 10 proteins were more abundant in swarming bacteria, as compared to two proteins more abundant in swimming bacteria.

**TABLE 3:** Flagellar activity associated proteins measured by LC-MS/MS.

**MS_experiment**			**EggNOG mapper**		**BlastKOALA**
**Locus_tag**	***p*-value**	**FC (Swarm – Swim) log2 difference**	**Predicted gene name**	**HMM_model_annotation**	**KEGG Orthology**
WP_080561184.1	0.08	5.65	YVYF	flagellar protein	NA
WP_016820950.1	0.01	5.46	FLII	Flagellum-specific ATP synthase	K02412
WP_016822917.1	0.15	5.3	FLGM	Anti-Sigma-28 factor, FlgM	K02398^∗^
WP_013368736.1	0.01	3.87	MOTA	MotA TolQ exbB proton channel	K02556
WP_019686987.1	0.06	3.55	FLIH	Flagellar assembly protein	K02411
WP_016820968.1	0.04	2.99	FLIA	D4	K02405
WP_013370683.1	0	2.46	FLBD	flagellar FlbD family protein	K02385
WP_016820955.1	0	2.3	FLGE	flagellar	K02390
WP_019686993.1	0.01	2.27	FLHF	flagellar biosynthesis regulator FlhF	K02404
WP_016820948.1	0.07	2.1	FLIF	D2	K02409
WP_019688794.1	0	2.08	FLIW	D6	K13626
WP_017427929.1	0.01	2.03	FLIZ/FLIO	flagellar	K02418
WP_019688791.1	0.01	1.78	FLIC	Flagellin	K02406
WP_019686456.1	0.16	1.71	NA	NA	K02410^∗^
WP_016820963.1	0	1.63	FLHA	Flagellar biosynthesis protein flha	K02400
WP_013370674.1	0.12	1.21	FLIG	D1	K02410
WP_016820957.1	0.45	1.13	FLIY	Flagellar motor switch protein	K02417
WP_013368735.1	0.21	0.91	MOTB	ompa motb domain protein	K02557
WP_016820956.1	0.23	0.83	FLIM	D3	K02416
WP_013370684.1	0.08	0.59	FLIL	flagellar basal body-associated protein	K02415^∗^
WP_016822895.1	0.89	0.18	FLGG	flagellar basal-body rod protein FlgG	K02392
WP_019688798.1	0.75	0.1	YVYF	flagellar protein	NA
WP_019688796.1	0.93	−0.03	FLGK	flagellar hook-associated protein	K02396
WP_019688795.1	0.74	−0.32	FLGL	flagellar hook-associated protein	K02397
WP_019688789.1	0.87	−0.46	FLID	flagellar hook-associated	K02407
WP_019686986.1	0.85	−0.68	FLGB	D5	K02387
WP_019688788.1	0.68	−1.11	FLIS	flagellar protein FliS	K02422
WP_016821367.1	0.21	−1.26	NA	NA	K02396^∗^
WP_019688790.1	0.06	−1.57	FLAG	flagellar protein FlaG	K06603
WP_019688756.1	0.46	−1.59	MOTA	MotA TolQ exbB proton channel	K02556
WP_029514992.1	0.55	−1.78	FLIR	D7	K02421
WP_019686118.1	0.21	−2.35	NA	NA	K02409^∗^
WP_007430020.1	0.09	−4.38	FLIE	flagellar hook-basal body complex protein fliE	K02408
WP_019687772.1	0.04	−6.16	NA	NA	K02390^∗^
WP_007430038.1	0.01	−6.39	FLIQ	Flagellar biosynthetic protein fliq	K02420

*^∗^ - Second best annotation of BlastKOALA; NA – annotation not predicted. Gene YVYF is an uncharacterized protein which may be involved in the assembly, structure, or function of the flagellum. May polymerize to form a filamentous structure that is part of the flagellum (https://www.uniprot.org/uniprot/P39807). D1: FliG is one of three proteins (FliG, FliN, FliM) that forms the rotor-mounted switch complex (C ring), located at the base of the basal body. This complex interacts with the CheY and CheZ chemotaxis proteins, in addition to contacting components of the motor that determine the direction of flagellar rotation (by similarity). D2: The M ring may be actively involved in energy transduction (By similarity) D3: FliM is one of three proteins (FliG, FliN, FliM) that forms the rotor-mounted switch complex (C ring), located at the base of the basal body. This complex interacts with the CheY and CheZ chemotaxis proteins, in addition to contacting components of the motor that determine the direction of flagellar rotation (By similarity). D4: Sigma factors are initiation factors that promote the attachment of RNA polymerase to specific initiation sites and are then released (By similarity). D5: Structural component of flagellum, the bacterial motility apparatus. Part of the rod structure of flagellar basal body (By similarity). D6: Binds to the C-terminal region of flagellin, which is implicated in polymerization, and participates in the assembly of the flagellum (By similarity). D7: Role in flagellar biosynthesis (By similarity).*

Previous studies have shown the importance of some flagellar proteins in the context of swarming motility. In this study, these highly abundant flagellar proteins in swarming motility also corroborates their importance in swarming bacteria. Some of the key flagellar proteins are evaluated below. Flagellin, FliC, is the basic subunit that polymerizes to form a helical flagellar filament (Kuwajima et al., 1986). Swarming in undomesticated *B. subtilis* was disrupted in a hag/flagellin mutant that was deficient in flagellum biosynthesis (Kearns and Losick, 2003). WP_019688791.1, annotated as Hag/FliC (K02406), is significantly more abundant (FC = 3.5×) in swarming bacteria relative to swimming bacteria. This is also the case for other major flagellar proteins, including FliO, FliI, and FliQ, which secrete flagellar structural components via a Type-III secretion system, and FliA, FliC, and MotA, which are the other major components of the flagella regulon. FliA, for example, regulates the expression of genes involved in flagellin production, initiation of flagellar filament assembly and length control, chemotaxis regulation, motor rotation, FliA-specific anti-sigma factor expression (Dasgupta et al., 2003), and swarming motility in *P. aeruginosa* (Lo et al., 2016). A homolog of FliA, SigD controls and governs the swarming motility in *B. subtilis* (Kearns et al., 2004). It is likely that FliA plays a similar role in swarming *P. polymyxa*.

**FIGURE 4:**
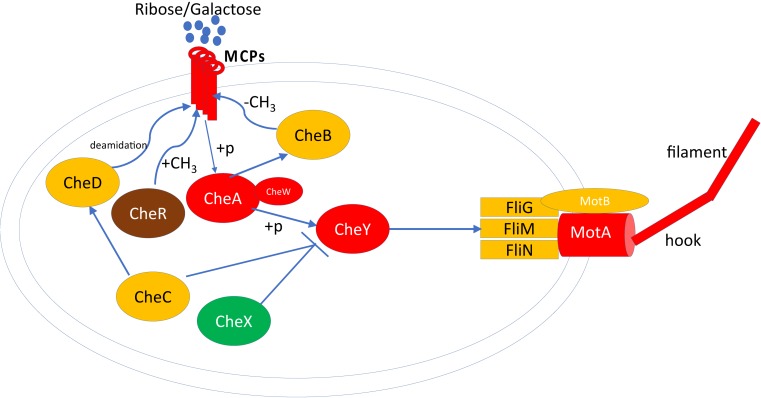
Proteomics measured proteins involved in chemotaxis and flagellar assembly in *P. polymyxa*. Only the proteins measured and detected by LC-MS/MS are considered in this diagram. The red color indicates the significantly higher abundance of protein in swarming motility, green color indicates significantly higher abundance of protein in swimming motility, yellow color indicates no significant difference in protein abundance in both motility and brown color indicate the multiple copies of protein detected significant in both motilities. MCP, methyl-accepting chemotaxis proteins; A, B, C, D, R, W, X, and Y are Che proteins; +p, phosphorylation.

A recent study revealed that the previously uncharacterized protein SwrD, encoded by the *ylzI* gene in *B. subtilis*, promotes swarming by increasing power to flagellar motors (Hall et al., 2017). A SwrD mutated *B. subtilis* strain abolished swarming but did not affect surfactant production or flagella production. However, overexpression of MotA and MotB restored swarming motility, suggesting SwrD’s role in increasing power to flagella motors. Blastp revealed that SwrD is 87% similar and 47% identical to WP_013370683.1 in *P. polymyxa* ATCC 842 and is also annotated as flagellar protein FlbD. This protein was significantly more abundant (∼6×) in swarming bacteria, suggesting a similar involvement in flagellar power-related activity. Previous findings have revealed that *Escherichia coli* and *Salmonella* need an additional protein, FliL, for swarming, which allows flagella to generate higher torque when grown on a surface (Motaleb et al., 2011). To this end, flagellar protein FliL from *B. subtilis* was queried against *P. polymyxa* ATCC 842, leading to the identification of ortholog WP_013370684.1 (97% query coverage and 37% sequence identity), which consists of a conserved FliL domain identified by three different databases. FliL was high in abundance but unchanged across growth conditions. FliG, FliN, and FliM form the flagellar rotor-mounted switch complex (C ring) located at the base of the flagellar basal body. This complex interacts with the CheY chemotaxis proteins, is regulated by the other components of the chemotaxis system (Spohn and Scarlato, 2001), and is directly involved in switching the direction of flagellar motor rotation upon CheY∼P binding (Figure 4). As demonstrated, CheA, CheW, CheY, M, and MotA were found to be significantly more abundant in swarming bacteria as compared to swimming bacteria. Interestingly, multiple copies of CheR exist in *P. polymyxa*; CheR_2 (WP_017425950.1) was 4–5× higher in swarming bacteria, in line with RT-PCR results as shown in Figure 5, whereas CheR_1 (WP_013371673.1) was 3–4× higher in swimming bacteria. It was interesting to observe two proteins annotated as CheR with methyltransferase activity, especially as their abundance profiles countered one another, and possibly suggest that each has a specific regulatory role directing these two modes of bacterial motility.

### Swarming *P. polymyxa* Upregulate a Non-ribosomal Peptide Biosynthetic Gene Cluster That Generates Surfactants and Polyketides

Non-ribosomal peptide synthetases generate many natural products with diverse biological activities, including lipopeptides that demonstrate antifungal, surfactant, and antibiotic properties (Choi et al., 2008;Li and Jensen, 2008; Roongsawang et al., 2010). The draft genome sequence of *P. polymyxa* ATCC 842 consists of a repertoire of biosynthetic genes for antibiotics (Jeong et al., 2011). Other strains of *P. polymyxa* have also been queried for potential lipopeptide antibiotic production using bioinformatics approaches (Choi et al., 2008; Kim et al., 2010; Ma et al., 2011; Niu et al., 2011; Huang and Yousef, 2012). Swarming bacteria are known to secrete extracellular wetting agents or biosurfactants to reduce surface tension between the substrate and the bacterial cell; this study reveals that *P. polymyxa* is quite similar. Sixteen different enzymes detected by LC-MS/MS were annotated as polyketide synthase (Pks), a multienzyme complex that can synthesize biosurfactants via successive condensation of small carboxylic acids (Gaisser and Hughes, 1997). Out of these, 14 enzymes are encoded by genes located in the same operon (3740422–3804256 bp), and 10 of these enzymes showed significantly increased abundance in swarming bacteria, indicating the importance of polyketides to swarming motility (Table 4). WP_016822428.1, annotated as PksD (polyketide biosynthesis acyltransferase), was detected only in swarming bacteria in all replicates. According to KEGG Brite, the Pks cluster (E, D, J, L, M, N, and R) encodes bacillaene (Kanehisa and Goto, 2000; Stein, 2005). In this study, genes encoding Pks (H, J, and L) and acyltransferase (D) proteins were investigated for gene expression using RT-PCR (Figure 5). The expression of *pksH* was seen only in swarming bacteria, whereas *pksJ* and *pksD* were significantly upregulated in swarming bacteria relative to swimming bacteria. Since almost the entire Pks cluster (Table 4), including bacillaene-encoding proteins, were upregulated during swarming conditions (as validated by both proteomics and RT-PCR measurements), it is likely that *P. polymyxa* produces a protein similar to bacillaene during swarming motility. Previously, a Δ*bae* mutant strain of *B. subtilis* incapable of producing bacillaene was determined to be deficient in swarming (Gao et al., 2017), which suggests that the *pks* gene cluster may be essential for swarming *P. polymyxa*.

**TABLE 4:** Proposed bacillaene PKS module in *P. polymyxa*.

**Protein**	***p*-value**	**FC (Swarm-Swim) log2**	**Gene start**	**Gene end**	**Gene (Prokka)**	**EC number**	**Protein description**
WP_019687989.1	0.46	−0.68	3740422	3742773	pksE		Polyketide biosynthesis protein PksE
WP_016822428.1	0.00	6.34	3742805	3743758	pksD	2.3.1.-	Polyketide biosynthesis acyltransferase PksD
WP_016822429.1	0.02	3.62	3743821	3744696	sadH	1.-.-.-	Putative oxidoreductase SadH
WP_019687990.1	0.00	3.67	3744756	3753674	pksN_2	2.3.1.-	Polyketide synthase PksN
WP_019687991.1	0.00	2.90	3753706	3767112	pksM		Polyketide synthase PksM
WP_019687992.1	0.00	2.53	3767163	3786932	pksL	NA	NA
WP_049789246.1	0.00	2.18	3787775	3797348	pksJ		Non-ribosomal peptide synthase
WP_016821481.1	0.01	1.43	3797581	3798330	pksI	4.-.-.-	Putative polyketide biosynthesis enoyl-CoA isomerase PksI
WP_019687993.1	0.03	3.72	3798377	3799138	pksH	4.2.1.-	putative polyketide biosynthesis enoyl-CoA hydratase PksH
WP_016821483.1	0.09	1.36	3799135	3800397	pksG	2.3.3.-	Polyketide biosynthesis 3-hydroxy-3-methylglutaryl-ACP synthase PksG
WP_019687994.1	0.08	3.05	3800413	3801660	pksF	4.1.1.87	Polyketide biosynthesis malonyl-ACP decarboxylase PksF
WP_016821485.1	0.00	2.88	3801638	3801877	acpK	NA	Polyketide biosynthesis acyl-carrier-protein AcpK
WP_019687995.1^∗^	0.02	1.80	3801903	3802664	NA	NA	hypothetical protein
WP_019687996.1	0.13	−1.77	3803621	3804256	baeB	3.-.-.-	putative polyketide biosynthesis zinc-dependent hydrolase BaeB

*^∗^Predicted as 4′-phosphopantetheinyl transferase by HMMER. Required for cells of *B. subtilis* to become producers of the lipopeptide antibiotics surfactin and plipastatin B1 (https://www.uniprot.org/uniprot/P39135).*

Besides Pks, lantibiotic biosynthesis proteins (WP_019686237.1, WP_017426262.1), annotated as NisB and NisC proteins, respectively, were more abundant in swarming bacteria. Lantibiotics commonly function as pheromones in quorum-sensing in *B. subtilis* (Stein, 2005). The higher abundances of these biosynthetic gene clusters in swarming bacteria implies roles in signaling, surfactant production, and antimicrobial activity. These antimicrobial/non-ribosomal peptides also are known to target phospholipids and alter membrane properties, such as intrinsic membrane curvature and fluidity (Ernst and Peschel, 2011; Epand et al., 2016) and could be intrinsic to membrane remodeling processes that are driven by different growth conditions as indicated by observed lipidomic differences in phospholipid composition.

### Surfactin Enhances the Swarming Capability of *P. polymyxa*

Besides Pks, surfactin has been proven vital for swarming motility in Gram-positive bacteria (Ohgiwari et al., 1992; Wakita et al., 1998; Julkowska et al., 2005). Swarming *B. subtilis* are known to produce surfactin, which enhances the level of CL in the membrane (Seydlová et al., 2013). Interestingly, the protein responsible for surfactin production is significantly more abundant in swarming *P. polymyxa*. In particular, SrfAA_ 4 (WP_019687302.1), surfactin synthase subunit 1, was significantly higher in swarming bacteria (∼3×) when compared to swimming bacteria. Increased expression of WP_019687302.1 in swarming bacteria, as measured by RT-PCR (Figure 5A), corroborates the proteomic data. In order to further examine the role of surfactin on swarming motility in *P. polymyxa*, swarming assays on agar plates were conducted with varying concentrations of surfactin. Compared to an untreated control, the addition of surfactin to the agar plate dramatically increased swarming motility (Figure 5B), which re-emphasizes the important role surfactant plays in swarming behavior in general, but also as it pertains to *P. polymyxa*.

## Conclusion

Despite significant advances in sequencing complete bacterial genomes, many studies of bacterial phenotypes still are restricted to macroscopic physiology characterizations. For example, coordinated bacterial motility, such as swarming, has been systematically studied, but an investigation into the detailed molecular machinery, in particular connecting lipids and proteins/enzymes, that comprise this unusual and environmentally important phenotype has not been done prior to this study. To address this deficit, an integrated lipidomic and proteomic measurement approach was designed to unravel the molecular level details of how swimming and swarming phenotypes differ in *P. polymyxa* ATCC 842. Despite the availability of a sequenced genome, not much is known about the physiology of this organism, mainly because *Paenibacillus* is notoriously recalcitrant to molecular genetics. This study reveals several novel findings, such as utilization of the pentose phosphate pathway and upregulation of glycine cleavage in swarming bacteria. Additionally, two modes of glycerol utilization (biosynthesis of phospholipids and utilization of glycerol via glycolysis/pentose phosphate pathway) were found. In particular, swarming bacteria revealed highly abundant proteins that degrade phospholipids to create sn-G3P and feed it into the glycolysis/pentose phosphate pathway for energy generation. Thus, energy generation through this mechanism appears likely to be necessary for swarming motility, including flagellar rotation. This study also revealed fine-level details of how the phospholipid composition is tuned to a particular phenotype. In particular swarming bacteria accumulate PA, while swimming bacteria favor LysoPA accumulation. Potential roles for glycosyl hydrolases and specialized flagellar activity and chemotaxis emerged from detailed proteomics measurements. An increased abundance of biosynthetic gene clusters indicated that surfactant was critical for *P. polymyxa* swarming. While some of the genes/proteins related to above-mentioned pathways were expected from the macroscopic physiology measurements, the integrated omics study in this report provided a high-resolution inventory of the specific lipids and proteins/enzymes that are connected to coordinate bacterial motility.

## Data Availability Statement

The datasets generated for this study can be found in the all raw mass spectra for the proteome measurements have been deposited into the ProteomeXchange repository with the following accession numbers: (MassIVE Accession: MSV000083145 and ProteomeXchange: PXD011747, FTP link to files: ftp://MSV000083145@massive.ucsd.edu, Reviewer password: “abcd1234”).

## Author Contributions

RH, JM-F, and JE designed the project and selected the microbial organism and phenotypes of interest. SP performed all the proteome measurements, associated the data-mining/analyses, and integrated the lipidomics and proteomics. AF and SC performed the lipidomics work. AB, JM-F, and JE designed the cell growth, cultivation, and RT-PCR work. RG provided technical advice and assistance on the proteomics work. SP and RH were primarily responsible for manuscript construction, with input/edits from all co-authors. All authors have inspected and approved the final submitted version.

## Disclaimer

This manuscript has been authored by UT-Battelle, LLC under Contract No. DE-AC05-00OR22725 with the United States Department of Energy. The United States Government retains and the publisher, by accepting the article for publication, acknowledges that the United States Government retains a non-exclusive, paid-up, irrevocable, world-wide license to publish or reproduce the published form of this manuscript, or allow others to do so, for United States Government purposes. The Department of Energy will provide public access to these results of federally sponsored research in accordance with the DOE Public Access Plan (http://energy.gov/downloads/doe-public-access-plan).

## Conflict of Interest

The authors declare that the research was conducted in the absence of any commercial or financial relationships that could be construed as a potential conflict of interest.
