# Supplementary material for: Integrated Proteomics and Lipidomics Reveal That the Swarming Motility of Paenibacillus polymyxa Is Characterized by Phospholipid Modification, Surfactant Deployment, and Flagellar Specialization Relative to Swimming Motility
Source: Front Microbiol. 2019 Nov 19;10:2594. doi: 10.3389/fmicb.2019.02594 (PMC6878767; doi:10.3389/fmicb.2019.02594)
Supplement: Supplementary file 1 [file Data_Sheet_1.docx]

Additional Materials for

**Integrated proteomics and lipidomics reveal that the swarming motility of *Paenibacillus polymyxa* is characterized by phospholipid modification, surfactant deployment, and flagellar specialization relative to swimming motility**

^†^ Suresh Poudel^2,3^; Richard J. Giannone^1^; Abigail T. Farmer^4,5^; Shawn R. Campagna^4,5^; Amber N. Bible^2,6^; Jennifer L. Morrell-Falvey^2,3,6^; James G. Elkins^2^; and Robert L. Hettich^1,3^*

^1^Chemical Sciences Division; ^2^Biosciences Division, Oak Ridge National Laboratory, Oak Ridge, TN, ^3^Graduate School of Genome Science and Technology, University of Tennessee, Knoxville, TN, ^4^Department of Chemistry, University of Tennessee, Knoxville, TN, ^5^Biological and Small Molecule Mass Spectrometry Core, University of Tennessee, Knoxville, TN, ^6^Department of Biochemistry & Cellular and Molecular Biology, University of Tennesssee, Knoxville, TN.

Corresponding author: Robert L. Hettich, Oak Ridge National Lab, Oak Ridge, TN 37831

Email: [hettichrl@ornl.gov](mailto:hettichrl@ornl.gov), Phone: 865-574-4968

This manuscript has been authored by UT-Battelle, LLC under Contract No. DE-AC05-00OR22725 with the U.S. Department of Energy. The United States Government retains and the publisher, by accepting the article for publication, acknowledges that the United States Government retains a non-exclusive, paid-up, irrevocable, world-wide license to publish or reproduce the published form of this manuscript, or allow others to do so, for United States Government purposes. The Department of Energy will provide public access to these results of federally sponsored research in accordance with the DOE Public Access Plan (<http://energy.gov/downloads/doe-public-access-plan)>.

**Supplementary Figure 1**. Ceramide metabolism: All the enzymes shown were measured by LC-MS/MS. The red-colored enzymes are significantly higher in abundance in swarming motility and black-colored enzymes are unchanged in abundance when swarming is compared to swimming.


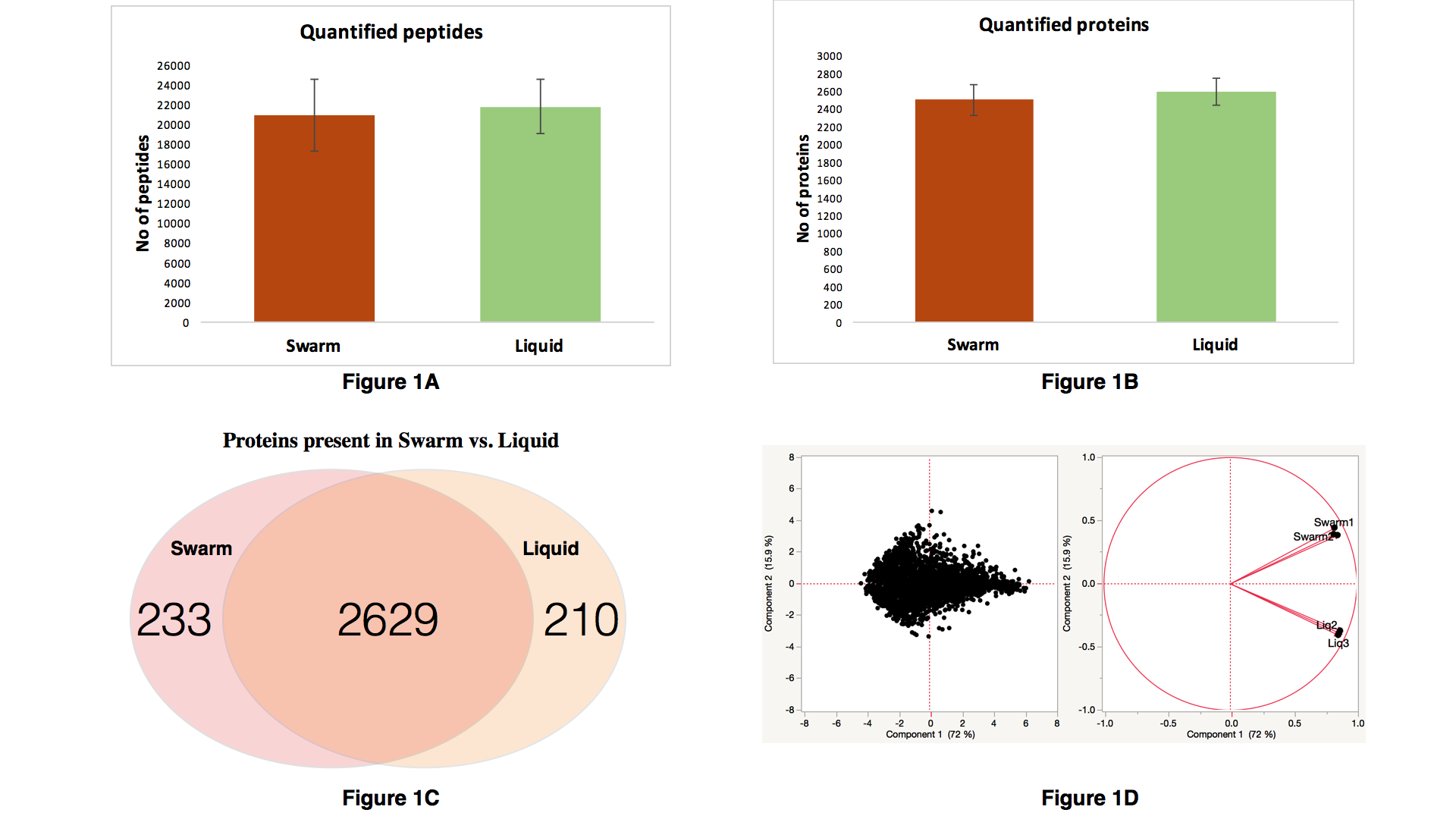


**Supplementary Figure 2.** Global proteome measurements in swimming and swarming *P. polymyxa*: A. Total number of quantified peptides; B. Total number of quantified proteins; C. Venn-diagram showing the total overlap of proteins and total unique proteins in swimming and swarming cultures; D. Visualization of protein distribution using PCA plot. Liquid – swimming motility, Swarm – swarming motility
